# Eosinophilic esophagitis: novel concepts regarding pathogenesis and clinical manifestations

**DOI:** 10.1007/s00535-019-01604-7

**Published:** 2019-07-24

**Authors:** Stuart Jon Spechler

**Affiliations:** 1 Division of Gastroenterology and Center for Esophageal Diseases, Baylor University Medical Center, 3500 Gaston Avenue, 2 Hoblitzelle, Suite 250, Dallas, TX 75246 USA; 2 Center for Esophageal Research, Baylor Scott & White Research Institute, Dallas, TX USA

**Keywords:** Proton pump inhibitors, Achalasia, Esophageal motility

## Abstract

This report explores two hypotheses regarding eosinophilic esophagitis (EoE): (1) that the use of proton pump inhibitors (PPIs) might contribute to the pathogenesis of EoE by preventing peptic digestion of food allergens, by increasing gastric mucosal permeability to enable gastric absorption of those undegraded food allergens, and by causing microbial dysbiosis, and (2) that EoE, like eosinophilic gastroenteritis, might have mucosal-predominant and muscle-predominant forms, and that the muscle-predominant form of EoE might cause a variety of esophageal motility disorders including achalasia.

## Introduction

Eosinophilic esophagitis (EoE) is a modern malady that was not recognized as a unique clinicopathologic syndrome until 1993 [1]. Since then, its frequency has soared, especially in Western countries such as the United States in which the prevalence of EoE (50–100 cases/100,000 persons) now is similar to that of ulcerative colitis [2–5]. EoE has become the most common cause of food impactions treated in emergency rooms [6], and EoE healthcare costs in the United States alone exceed $1 billion annually [7].

## Potential role of proton pump inhibitors in EoE pathogenesis

The cause of the recent appearance and dramatic rise in the frequency of EoE remain unknown. Proposed explanations include: [8] (1) the hygiene hypothesis, which holds that modern hygienic conditions have resulted in fewer childhood infections that would have protected against allergic disorders such as EoE, (2) modern western lifestyle changes and early life events have resulted in microbial dysbiosis (altered composition and diversity of the microbiome) that predisposes to EoE, (3) modern changes in environmental factors (e.g. genetic modifications of and chemicals added to crops, livestock treatment with hormones and antibiotics, food additives, food processing and packaging changes, air and water pollution) contribute to EoE pathogenesis, (4) *H. pylori* infection, which can induce regulatory T cells that might protect against EoE and other allergies, is declining in frequency, and (5) there is an increasing frequency of gastroesophageal reflux disease (GERD), which can impair esophageal mucosal integrity, thus enabling mucosal penetration of food antigens that trigger EoE. For gastroenterologists, perhaps the most intriguing hypothesis to explain the appearance and increasing incidence of EoE relates to the use of acid-suppressant medications, especially the proton pump inhibitors (PPIs) [9].

The rise in the frequency of EoE has closely paralleled the rising usage of PPIs over the past several decades [10]. While this association alone cannot establish cause and effect, the proposed mechanisms whereby PPIs might predispose to EoE (discussed below) seem quite plausible. PPIs are an established treatment for EoE, and it might seem paradoxical that these same agents have been implicated as pathogenetic factors. The mechanisms underlying the therapeutic effects of PPIs in EoE are not clear, but probably are related to control of any underlying GERD that might be exacerbating the condition, and to the ability of PPIs to inhibit Th2 cytokine-stimulated secretion of the potent eosinophil chemoattractant eotaxin-3 by esophageal epithelial cells [11, 12]. The proposed mechanisms whereby PPIs might predispose to EoE pathogenesis are entirely different.

Normally, acid produced by gastric parietal cells and pepsins produced by gastric chief cells begin the process of hydrolyzing food proteins. Consequently, ingested proteins that are potentially allergenic can be hydrolyzed in the stomach into harmless peptide fragments. Such peptic digestion requires acidic conditions, and pepsins have little proteinase activity at pH levels above 4.5 [13, 14]. When PPIs raise gastric pH to those levels, food allergens that normally would be digested in the stomach can remain intact. Furthermore, PPIs increase gastric mucosal permeability through mechanisms that remain unclear, potentially enabling gastric absorption of the undigested food allergens [15, 16]. Undegraded, allergenic peptides that escape gastric absorption also might induce an immune response when they reach the small intestine. In addition, PPIs might enable the survival of microorganisms that normally would be destroyed by gastric acid, causing microbial dysbiosis that might mediate mucosal inflammatory responses to facilitate the development of food allergies [17–19].

Data from animal and human studies support the concept that acid suppressing medications can predispose to food allergy. Untersmayr et al. [20] noted that caviar proteins are rapidly digested by pepsin at pH 2.0, but not at pH 5.0. When those investigators fed caviar to mice treated with antisecretory medications, the mice developed caviar-specific IgE antibodies, T cell reactivity and gastric eosinophilia. Similarly, mice that were treated with antisecretory medications and fed hazelnuts developed IgG1 antibodies to hazelnut, and some developed type I skin reactivity to hazelnut extract [21]. In a study of 152 patients treated with antisecretory medications for 3 months, 10% developed a rise in IgE antibody levels, and 15% developed new, food-specific IgE antibodies [22]. In another human study of 153 patients on acid-suppressant medications for 3 months, 5 (3.3%) developed hazelnut-specific IgE antibodies; 4 of those developed specific skin reactivity and 2 showed clinical signs of allergy to hazelnuts [21].

One brief report has challenged the hypothesis that PPIs predispose to EoE [23]. That report, which was a post hoc analysis of a study on the prevalence of EoE among patients undergoing upper gastrointestinal endoscopy at a military hospital [24], described no significant difference in the frequency of current PPI usage between patients with EoE (15/25 patients, 60%) and those without EoE (239/360 patients, 66%). There also was no significant difference in the prevalence of EoE among PPI users (15/252, 6%) and nonusers (10/131, 7.6%), and no increase in the prevalence of EoE in patients on high dose PPIs [23]. Although the authors concluded that their findings did not support a pathogenetic role for PPIs in EoE, their study cannot refute such a role for several reasons. Study subjects were patients having endoscopy for upper gastrointestinal symptoms, and most were taking PPIs. Even if PPIs played a role in EoE pathogenesis, there might well be no difference in the rate of current PPI usage between patients with and without EoE in such a symptomatic population. Furthermore, the investigators asked only about current PPI usage, not about remote exposures to PPIs that might have triggered the development of EoE.

Finally, the immature immune system of infants might be especially susceptible to dysregulation by medications like PPIs that affect food antigens and the microbiome, and there has been a dramatic increase in infant PPI exposure in this millennium. Between 2002 and 2009, there was an 11-fold increase in the number of new PPI prescriptions for pediatric patients under 12 months of age [25]. A recent case–control study exploring associations between early life factors (e.g. Cesarean delivery, antibiotic and acid suppressant use in infancy, breastfeeding, etc.) and the later development of EoE in children found that the use of acid suppressants during the first year of life was the strongest of all potential EoE risk factors studied (adjusted OR 7.41, 95% CI 4.00, 13.74) [26]. Even when the investigators restricted the case sample to children reporting EoE symptoms at age 3 or older (to minimize protopathic bias), there remained a strong association between PPI use in infancy and the later development of EoE (adjusted OR 6.05, 95% CI 2.55, 14.40). Further studies on the potential pathogenetic role of PPIs in EoE clearly are warranted.

## Potential role of EoE in esophageal motility disorders

**Fig. 1:**
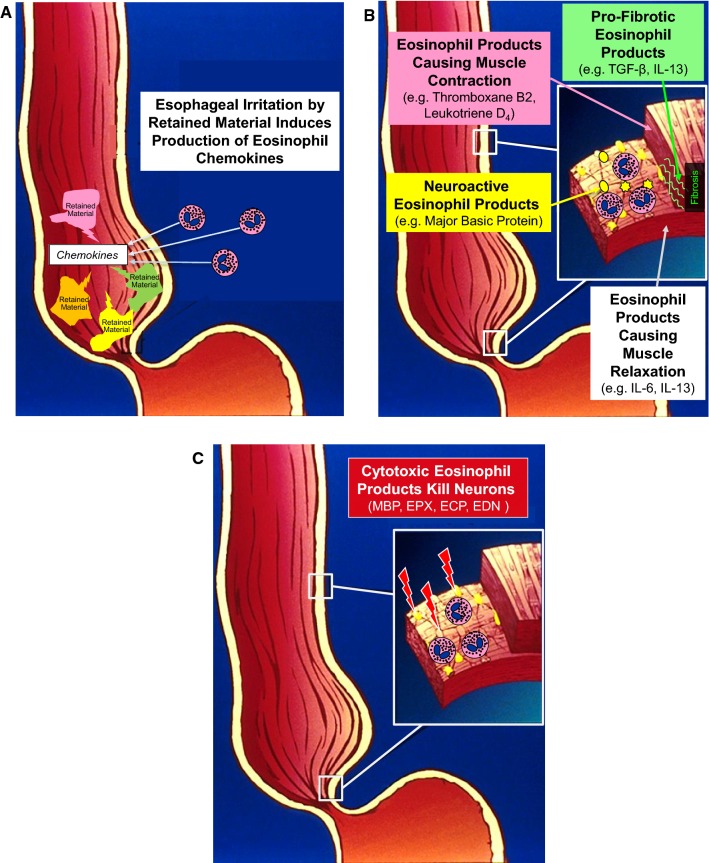
Potential mechanisms underlying the association of esophageal motility abnormalities and esophageal eosinophilia. **a** Primary esophageal motility abnormalities cause esophageal stasis with retained material that irritates the mucosa, inducing secretion of chemokines that attract eosinophils. **b** Motility abnormalities are caused by eosinophils in the esophagus that release myoactive, neuroactive, and pro-fibrotic eosinophil secretory products. **c** Motility abnormalities are caused by eosinophils in the esophagus that release cytotoxic eosinophil secretory products that destroy esophageal intramural neurons Modified illustration of esophagus and stomach used with permission, copyright, American Gastroenterological Association Institute, Bethesda, MD, and figure reproduced from reference 27 with permission from the American Journal of Gastroenterology

A variety of esophageal motility disorders, including achalasia, have been described in patients with EoE, and three potential mechanisms have been proposed to explain the association of motility abnormalities and esophageal eosinophilia (Fig. 1): [27] (1) The motility abnormalities are primary (i.e. not caused by esophageal eosinophils) and result in esophageal stasis with retained material that irritates the mucosa, inducing its secretion of chemokines that attract eosinophils. In this situation, treatments that improve esophageal emptying should improve mucosal eosinophilia. (2) The motility abnormalities are caused by eosinophils in the esophagus that release myoactive and neuroactive secretory products that disrupt motor function, and release pro-fibrotic products that induce tissue remodeling. In this situation, treatments that reduce esophageal eosinophilia should reverse motility abnormalities caused by myoactive and neuroactive eosinophil secretory products. (3) The motility abnormalities are caused by eosinophils in the esophagus that release cytotoxic secretory products that destroy the esophageal intramural neurons that mediate peristalsis and LES relaxation. In this situation, treatments aimed at esophageal eosinophilia might prevent further neuronal damage, but the motility abnormalities due to extant neuronal loss would be irreversible.

*Evidence that esophageal stasis causes mucosal eosinophilia*. In patients with motility disorders like achalasia that result in esophageal stasis, mucosal irritation caused by retained material in the esophagus might induce the secretion of chemokines that attract eosinophils. If so, then the esophageal eosinophilia should resolve with treatments that improve esophageal emptying. Although there are reports of patients with esophageal stasis due to achalasia who had esophageal mucosal biopsies showing dense eosinophilia [28], there are few data documenting the effect of achalasia treatment on esophageal eosinophilia. A study of 50 achalasia patients who had esophageal mucosal biopsies before and after Heller myotomy found that 17 (34%) had ≥ 1 eosinophil per high power field (hpf) in preoperative biopsies (median 3/hpf, range 1–21) [29]. Postoperatively, 6 of those 17 patients (35%) showed a decrease in esophageal eosinophilia (median 0.5/hpf, range 0–4), but 11 (65%) showed an increase (median 7/hpf, range 1–62). Furthermore, 11 of 33 patients (33%) with no epithelial eosinophils on preoperative biopsies showed intraepithelial eosinophilia postoperatively (median 3/hpf, range 1–15), and 4 with low-level eosinophilia preoperatively had dense eosinophilia postoperatively. Thus, there is little support for the concept that esophageal stasis causes EoE-level esophageal mucosal eosinophilia.

*Evidence that motility abnormalities are caused by eosinophils in the esophagus that release myoactive and neuroactive secretory products*. Eosinophils produce numerous cytokines, growth factors, and cationic proteins that can affect esophageal smooth muscle contractility [30]. Some eosinophil secretory products cause esophageal muscle to contract (e.g. leukotriene D_4_, prostaglandin F2 alpha, thromboxane B2) [31, 32], some cause its relaxation (e.g. interleukin [IL]-6, IL-13) [33], and some have disparate effects on esophageal muscle depending on experimental conditions (e.g. transforming growth factor [TGF]-β1) [33, 34]. Eosinophils also release neuroactive products that might influence esophageal motility. Eosinophil MBP potentiates the release of acetylcholine from parasympathetic nerves, and eosinophils can mediate changes in sensory nerve structure and neuropeptide expression [35]. In addition, eosinophils secrete pro-fibrotic products such as TGF-β, IL-13, IL-8, and vascular endothelial growth factor (VEGF) that can induce tissue remodeling, which might alter esophageal motility as it does in scleroderma [36]. Treatments that reduce esophageal eosinophilia should reverse motility abnormalities caused by myoactive and neuroactive eosinophil products, but tissue remodeling effects might be irreversible.

Since eosinophils secrete products that can either excite or relax esophageal muscle, esophageal eosinophilia might result in either hyper- or hypo-contractile motility disorders. Indeed, a variety of esophageal motility abnormalities have been described in patients with EoE including hypercontractile disorders such as nutcracker esophagus, hypertensive LES, jackhammer esophagus, and distal esophageal spasm; hypocontractile disorders such as ineffective esophageal motility, absent contractility, and hypotensive LES; and mixed (both hyper- and hypo-contractile) disorders such as pan-esophageal pressurization and achalasia [28, 37–47]. Hypocontractile abnormalities appear to be more frequent than hypercontractile abnormalities [42, 44]. Studies using high-resolution manometry have found esophageal motility abnormalities in 25–76% of EoE patients [43–47] with rates of motility abnormalities considerably higher in EoE patients than in healthy control subjects and/or patients with GERD [38, 40, 43–45].

As noted above, if motility abnormalities are caused by eosinophils in the esophagus that release myoactive and neuroactive secretory products, then those abnormalities should be reversible with treatments that reduce esophageal eosinophilia. Indeed, there are reports documenting normalization of esophageal motility disturbances in EoE patients treated with steroids [41, 47, 48]. Two reports describe patients with both EoE and achalasia who exhibited a return of esophageal peristalsis and LES deglutitive relaxation after steroid treatment had reduced their esophageal eosinophilia [41, 49]. These reports strongly suggest that eosinophils in the esophagus can cause motility disturbances that resolve with treatments that reduce eosinophil numbers.

*Evidence that motility abnormalities are caused by eosinophils in the esophagus that release cytotoxic secretory products that destroy intramural neurons*. The first report of an esophageal motility disorder associated with “eosinophilic esophagitis” was published in 1978 [50]. That report described a patient with achalasia who had biopsies of esophageal muscularis propria showing “heavy eosinophilic infiltration”, which the authors attributed to an unusual variant of eosinophilic gastroenteritis. The clinicopathologic syndrome that we now recognize as EoE was not described until 1993 [1, 3] and widespread recognition of this new disorder was delayed until well into the new millennium.

Achalasia is known to result from the loss of the esophageal intramural neurons that normally mediate peristalsis and deglutitive relaxation of the lower esophageal sphincter (LES) [51]. However, the cause of the esophageal neuronal degeneration of achalasia is not known. In 1989, investigators in Denmark described a patient who had achalasia associated with a gastric adenocarcinoma that was treated with gastrectomy [52]. Histologic examination of the resected distal esophagus revealed dense eosinophilia with immunohistochemical staining for eosinophil cationic protein (ECP) found in all layers of the esophageal wall. The investigators subsequently reported positive immunohistochemical staining for ECP in biopsies of esophageal muscularis propria taken during Heller myotomy in all of 9 patients with primary (idiopathic) achalasia [53]. Since ECP was known to be toxic to neurons, the investigators proposed that cytotoxic proteins released by degranulating eosinophils might cause achalasia by destroying esophageal intramural neurons.

In 1994, investigators from the University of Michigan reported finding eosinophils and lymphocytes infiltrating the myenteric plexus in all of 42 esophagectomy specimens resected from patients with end-stage achalasia, with eosinophilia involving the muscularis propria in 22 cases (52%) [54]. In 1996, a study of esophageal muscle biopsy specimens taken during Heller myotomy in 11 patients with early achalasia documented myenteric neuritis with T-lymphocyte-predominant inflammation in all cases, with a mixture of both lymphocytes and eosinophils found in 6 cases (55%) [55]. A recent study of 46 patients with achalasia or esophago-gastric junction outlet obstruction who had esophageal muscle biopsies taken during Heller myotomy found inflammatory infiltrates in 8 cases (17%), 7 of which were comprised predominantly of T-lymphocytes while 1 was predominantly eosinophils [56]. Since T lymphocyte subpopulations are involved in coordinating eosinophil influx into tissues during allergic responses, which appear to underlie the pathogenesis of EoE, the admixture of T lymphocytes and eosinophils found in achalasic esophageal muscle in these studies is especially noteworthy [57].

Like EoE, eosinophilic gastroenteritis is an eosinophilic gastrointestinal disorder (EGID) that appears to be immune/allergen mediated [58]. In 1970, Klein first proposed the now widely accepted clinicopathologic categorization of eosinophilic gastroenteritis that is based upon the layer of the gastrointestinal tract (mucosa, muscularis propria, or serosa) that exhibits the heaviest infiltration by eosinophils [59]. Mucosal-predominant eosinophilic gastroenteritis typically causes malabsorption and non-specific symptoms such as abdominal pain, nausea and diarrhea, while patients with muscle-predominant disease present with obstructive symptoms, and those with serosal-predominant disease develop eosinophilic ascites. EoE is diagnosed using esophageal mucosal biopsies that do not sample the muscularis propria, and so the Klein system has not been used to categorize EoE. Nevertheless, studies of rarely obtained esophagectomy specimens from EoE patients have found eosinophils infiltrating all layers of the esophageal wall including submucosa, muscularis propria and adventitia [33, 60]. It seems probable that EoE, like eosinophilic gastroenteritis, would have mucosal-predominant and muscle-predominant forms with different clinical manifestations, and esophageal motility abnormalities would be most likely to accompany EoE with muscular involvement.

In support of the concept that EoE might have a muscle-predominant form, Japanese investigators recently identified patients with hypercontractile esophageal motility abnormalities (jackhammer and nutcracker esophagus) who had no eosinophils in biopsies of the esophageal mucosa, but who had dense eosinophilic infiltrates in biopsies of esophageal muscularis propria taken during per-oral endoscopic myotomy (POEM) [48, 61]. Another study of 28 achalasia patients who had esophageal muscle biopsies taken during POEM found immunohistochemical staining for eosinophil major basic protein (MBP) and eosinophil-derived neurotoxin (EDN) in 24 cases (86%), and the authors speculated that achalasia with eosinophil infiltration of esophageal muscle might represent a subtype of EoE [62]. While these reports make it clear that patients can have esophageal motility disorders associated with eosinophils in the muscularis propria but not in the mucosa, it is not clear that the muscular eosinophil involvement in those patients is allergen-driven (i.e. a variant of EoE involving esophageal muscle but sparing the mucosa).

There also are case reports of patients with partial esophageal obstruction caused by eosinophils invading deep layers of the esophageal wall, but with esophageal biopsies showing only mild epithelial eosinophilia (< 5 eosinophils per high power field) [63, 64]. One patient had esophageal wall thickening so profound that esophagectomy was performed because he was presumed to have esophageal cancer [63]. Histologic evaluation of the resected esophagus revealed dense infiltration of eosinophils in the muscularis propria, especially around nerve bundles and ganglia. In another case of a woman with rapidly progressive dysphagia and weight loss, an esophageal endoscopic mucosal resection specimen showed dense eosinophilia in the lamina propria and submucosa, but the epithelium was spared [64]. That patient’s symptoms resolved completely when she was treated with systemic corticosteroids, and the authors concluded that she had isolated esophageal involvement by eosinophilic gastroenteritis rather than an EoE variant. However, the authors of an accompanying editorial commented that the distinction between EoE and eosinophilic gastroenteritis is not well delineated [65].

It has long been appreciated that eosinophils are effector cells that release toxic cationic proteins to kill microorganisms [66]. Some of those proteins also can kill human cells, including neurons. Eosinophil MBP destroys mammalian cells by disrupting the lipid bilayers of their membranes [67]. Eosinophil peroxidase (EPX) functions as a peroxidase when hydrogen peroxide is present, but EPX is a potent cationic cytotoxin fully capable of killing mammalian cells even in the absence of hydrogen peroxide [68]. Eosinophil cationic protein (ECP) and eosinophil-derived neurotoxin (EDN) have ribonuclease activity that is toxic to neurons [68]. Indeed, EDN was so named because it caused a neurotoxic reaction when injected into the brains of experimental animals. Thus, eosinophils infiltrating the esophageal muscle might degranulate and release toxic proteins, destroying neurons in the myenteric plexuses and thereby causing achalasia or other motility abnormalities. In addition to releasing toxic eosinophil cationic proteins, eosinophils have important antigen-presenting and immunomodulatory functions that conceivably could contribute to the destruction of esophageal neurons [69].

## Conclusions

Esophageal eosinophilia frequently is associated with a variety of hypo- and hyper-contractile esophageal motility disorders including achalasia, and available evidence suggests that esophageal stasis is not an important cause of esophageal mucosal eosinophilia. Many patients with achalasia have an abnormal accumulation of eosinophils and their degranulation products in the esophageal muscularis propria, a location inaccessible to routine endoscopic evaluation. Reports document that esophageal motility abnormalities can respond to steroid treatment that reduces esophageal eosinophilia, suggesting that eosinophils in the esophagus can cause reversible motility disturbances, perhaps by releasing myoactive and neuroactive eosinophil products. In addition, degranulating eosinophils release toxic proteins capable of destroying enteric neurons and, thereby, causing irreversible motility abnormalities. It seems likely that EoE, like eosinophilic gastroenteritis, might have mucosal-predominant and muscle-predominant forms that have different clinical manifestations, and esophageal motility abnormalities might well be a consequence of muscle-predominant EoE. Unfortunately, there is no simple, minimally-invasive way to obtain biopsies of esophageal muscle to confirm the presence of muscle-predominant EoE. Such biopsies require a laparoscopy or POEM procedure.

The concept that eosinophils in esophageal muscle might underlie motility disorders has important therapeutic implications. For one, it suggests that some motility disorders might respond to treatments that decrease muscular infiltration by eosinophils. Furthermore, for patients with esophageal motility disorders who are known to have EoE because they have esophageal mucosa eosinophilia, the medications commonly used to treat EoE might not affect the eosinophil-induced motility abnormalities. Topical steroids and proton pump inhibitors (PPIs) are aimed primarily at correcting mucosal eosinophilia. Although there are reports of topical steroids correcting motility abnormalities in patients with mucosal EoE [41, 47, 48], it is not clear how often these agents penetrate deeply enough to influence eosinophilia in the esophageal muscles. As mentioned, PPIs might reduce mucosal eosinophilia in EoE by inhibiting Th2 cytokine-stimulated release of an eosinophil chemoattractant (eotaxin-3) by esophageal epithelial cells [11, 12]. However, it has been reported that PPIs do not block Th2 cytokine-stimulated eotaxin-3 secretion by subepithelial esophageal fibroblasts [70], and the effects of PPIs on eotaxin-3 secretion by esophageal muscle are not known. Thus, PPIs that eliminate eosinophils from the mucosa might have little effect on eosinophilic infiltration of the submucosa and muscularis propria. Consequently, PPI therapy for EoE may appear effective by mucosal biopsy, while eosinophils might persist in deeper layers of the esophageal wall where they continue to induce motility abnormalities.

It is important to appreciate that the hypotheses presented in this report (that PPIs might predispose to EoE pathogenesis and that EoE has a muscle-predominant form that causes esophageal motility disorders) are not established. Nevertheless, these are plausible hypotheses, supported by considerable indirect evidence, and both have potentially important clinical implications. Further investigations on the role of PPIs in the pathogenesis of EoE, and on the role of eosinophils in the pathogenesis of esophageal motility disorders clearly are warranted and eagerly awaited.
